# Effects of Baby Schema and Mere Exposure on Explicit and Implicit Face Processing

**DOI:** 10.3389/fpsyg.2019.02649

**Published:** 2019-11-29

**Authors:** Leonardo Venturoso, Giulio Gabrieli, Anna Truzzi, Atiqah Azhari, Peipei Setoh, Marc H. Bornstein, Gianluca Esposito

**Affiliations:** ^1^Department of Psychology and Cognitive Science, University of Trento, Trento, Italy; ^2^Psychology Program, School of Social Sciences, Nanyang Technological University, Singapore, Singapore; ^3^Trinity College Institute of Neuroscience, Trinity College Dublin, Dublin, Ireland; ^4^Institute for Fiscal Studies, London, United Kingdom; ^5^National Institute of Child Health and Human Development, Bethesda, MD, United States

**Keywords:** baby schema, mere exposure effect, face processing, neural oscillations, pupillometry, EEG

## Abstract

In an increasingly multicultural society, the way people perceive individuals from the same vs different ethnic groups greatly affects their own and societal well-being. Two psychological effects that influence these perceptions are the Mere-Exposure Effect (MRE), wherein familiarity with certain objects or persons suffices for people to develop a preference for them, and the Baby Schema (BS), a set of specific facial features that evokes caregiving behaviors and an affective orientation in adults. In the present study, we aimed to investigate whether these two effects play a role in implicit physiological responses to babies vs. adults faces belonging to participants in-group vs. out-group. In study 1, the pupillary diameter of 62 Caucasian participants (M = 31; F = 31) who observed adult and infant faces of different ethnic groups (Caucasian, Chinese) was measured. In study 2, brain waves of 38 Caucasian participants (M = 19; F = 19), who observed the same set of faces, were recorded using EEG. In both studies, adults explicit preferences (i.e., attitudes) toward faces were assessed using questionnaires. In Study 1, females showed greater attention to infant than adult faces (BS effect) in both pupils, regardless of the ethnic group of the face. By contrast, males attended to infant more than adult faces for out-group faces only (BS effect). In Study 2, greater left posterior-parietal alpha activation toward out-group compared to in-group adult faces was found in males (MRE). Participants with a low BS effect toward in-group baby faces exhibited greater left posterior alpha activation to out-group than in-group baby faces (MRE). These findings reveal how different levels of sensitivity to in-group infants may moderate perceptions of both in-group and out-group baby faces. Questionnaire measures on attitudes showed that males and females preferred in-group to out-group adult faces (MRE). Participants in Study 2 also reported a greater preference for infants than adults faces (BS effect). These findings explicate the roles of gender and the Baby Schema effect in moderating implicit processing of in-group and out-group faces, despite their lack in moderating explicit reports. Contradictory findings at the implicit (physiological) and explicit (self-report) levels suggest that differential processing of faces may occur at a non-conscious level.

## 1. Introduction

Faces are central in social cognition. From a person's face, we efficiently extract an immense amount of information (e.g., age and ethnicity) that guide our behavioral responses (Willis and Todorov, 2006). Of particular salience is the infant's face, which is crucial in the context of parenting. It is well-known that rapid and appropriate responses to infants needs enable the wholesome development of social, cognitive, and emotional domains, whereas incoherent responses lead to adverse developmental trajectories (Bowlby, 1969; Waters et al., 2000; Furman and Buhrmester, 2009; Bornstein, 2015). Adults parental responses have been shown to be facilitated by a rapid shift in attention toward infants faces. As compared to faces of adults and non-human infants/adults, faces of human infants command greater levels of attention (Brosch et al., 2007). The automatic orientation to an infant's face suggests that it serves as a cue to trigger a distinct set of brain responses that promotes adults adaptive caregiving (Plutchik, 1987; Seifritz et al., 2003).

Baby Schema refers to the set of physical and behavioral characteristics typical of babies (such as big round eyes, big head, and small face, small ears, short limbs, clumsy gait) that evoke protective caregiving behaviors in adults (Lorenz, 1943; Lorenz and Martin, 1971). The Baby Schema effect is more evident in women than in men, suggesting gender differences in processing infant faces (Baron-Cohen et al., 2003; Glocker et al., 2009a). However, little is known of whether and how the familiarity of ethnic in-group facial cues, as compared to less familiar out-group features, moderates adults responses to infant faces. The Mere Exposure Effect is a phenomenon by which increased exposure to an object or person leads to enhanced familiarity and contributes to developing a preference (Zajonc, 1968). For instance, Bornstein and Tamis-LeMonda (1989) found that a wide range of affective responses increases with exposure (e.g., pleasantness, liking, etc.), and this effect is applicable to a variety of stimuli (e.g., abstract paintings, polygons, line drawings, etc.), including faces. The Mere Exposure Effect suggests that preference for faces would increase to familiar in-group adult and infant faces. However, it remains to be seen whether the protective effect of Baby Schema would lead to an increased preference for infant faces regardless of whether the face belongs to an in-group (familiar) or out-group (unfamiliar). Preferences for facial stimuli can be measured in many ways, such as by recording autonomic physiological responses. Pupillometry assesses pupillary response to emotionally relevant visual stimuli.

Dilation of the pupil indicates physiological arousal, reflecting the activation of the sympathetic branch (i.e., arousal response) of the autonomic nervous system toward visual stimuli (Hess and Polt, 1960; Aktar et al., 2016; Wetzel et al., 2016). In general, larger pupillary dilations are evoked by more pleasant or more aversive stimuli (Steinhauer, 1983; Bradley et al., 2008). On the other hand, pupil constriction happens when the circular muscle contracts, reflecting the activation of the parasympathetic branch (i.e., calming response) of the autonomic nervous system. However, the interpretation of the meaning of pupil constriction is not completely clear and unambiguous. In fact, while Loewenfeld (1966) argued that the only factor causing pupil constriction is the increased light intensity and while Hess (1972) suggested that constriction occurs in response to unpleasant or distasteful visual stimuli, other recent studies (Mathôt et al., 2013; Mathôt and Van der Stigchel, 2015; Landi and Freiwald, 2017; Winn et al., 2018; Zekveld et al., 2018) underlined that distinct cognitive processes, including attentional, emotional and motivational ones, may differently intervene in mediating the strength of pupillary constriction. For example, Landi and Freiwald (2017) found that greater constrictions occur in response to familiar stimuli than unfamiliar ones. The pupillometry has been used in order to study several variables involved in face processing (i.e., age, ethnic group, and gender). Regarding the ethnic group, differential responses to in-group and out-group are reflected in pupil dilation and constriction. For instance, Goldinger et al. (2009) and Wu et al. (2012) found that viewers pupil size was larger when processing out-group than in-group faces, meaning that more attention is given to out-group faces. Moreover, Landi and Freiwald (2017) found that viewers pupil size decrease considerably when processing familiar faces compared to unfamiliar ones, suggesting that familiar faces request fewer cognitive resources in order to be processed. These studies show that the Mere Exposure Effect is reliably observed using measures of pupillometry.

Another variables that have been investigated are gender and age. In accordance with known gender effects of Baby Schema, women display relatively larger pupillary dilation, which means greater attention to certain stimuli, when they view pictures of infants than people of other ages (Hess and Polt, 1960). To our knowledge, no studies have found larger pupillary responses for adult faces compared to baby faces in female participants. Thus, the Baby Schema effect is sensitively captured by pupillometry.

Several studies (Andrew et al., 1982; Hamilton and Vermeire, 1988; Prior et al., 2002; Regolin et al., 2004) have also found that the pupillary response may variate between pupils and that this laterality may indicate differentiated processing of stimuli. For instance, Andrew et al. (1982) found that the left eye of the domestic chick is more reactive to emotional stimuli compared to the right one. Hamilton and Vermeire (1988) also claimed that the domestic chick's right eye is involved in foraging behavior, while the left one is engaged in alert behavior. Moreover, Prior et al. (2002) and Regolin et al. (2004) have proved that the left eye and its contralateral connections to the right hemisphere are relevant in the processing of spatial information.

A more complete understanding of physiological responses requires however measures of both peripheral and central nervous systems. Pupillometry captures the former, but electroencephalography (EEG) records brain oscillatory activity as an index of the latter. Asymmetrical alpha band oscillations (8–12 Hz) indicate facial preferences (Kang et al., 2015). This asymmetrical pattern is elicited if stimuli are especially arousing, regardless of their emotional valence (Balconi and Mazza, 2009). Investigations using infant stimuli have revealed that electrophysiological activity toward infants faces is greater in women than in men (Proverbio et al., 2011). Proverbio and De Gabriele (2017) also showed that there was no observable difference in electrophysiological activity when viewing infant faces of different ethnicities, lending support to the wide application of protective effects of Baby Schema. EEG has also been used to examine the Mere Exposure Effect. Thiruchselvam et al. (2016) showed that increased exposure to infant faces affects attractiveness ratings and posterior neural reactivity. In a study using in-group and out-group faces, Zheng and Segalowitz (2014) learned that group membership influences brain electrical activity specific to faces. Thus, in addition to pupillometry, we used EEG to investigate both Baby Schema and Mere Exposure Effects. Despite past extensive investigation on this topic, no study has considered how responses to in-group baby faces and out-group baby faces are associated, and how the processing of faces in each of these categories may be moderated by participants gender.

The present study aimed to investigate Baby Schema and Mere Exposure Effects on adults peripheral and central nervous system and attitudinal responses to infant and adult faces of ethnic in-group and out-group. We conducted two studies: Study 1 used pupillometry in Caucasians (*N* = 62), and Study 2 recorded EEG in Caucasians (*N* = 38). In both studies, participants were presented with Chinese and Caucasian adults and infants faces. Alongside implicit neurophysiological measures, adults explicit attitudes to faces were assessed using a questionnaire. Several studies, including Glocker et al. (2009a,b) and Caria et al. (2012) focused on how BS activates the neural system of both females and males, while others, such as Esposito et al. (2015) explicitly compared emotional responses in the two genders. However, as far as we know, no study has taken into account an additional variable which is the familiarity of faces (in-group vs. out-group) to investigate how BS influences face processing. The Life History Theory (LHT) highlights that males and females of different species exhibit distinct attitudes toward babies and parental activities (Draper and Harpending, 1982; Mascaro et al., 2013). They adopt distinct strategies to optimize their fitness: parenting and mating. As females place more resources in reproduction (for example for pregnancy and labor), evolution motivates her to engage in parenting/caregiving activities that improve the survival and quality of the offspring. Indeed, it seems that higher rates of reproduction in humans are associated with lower offspring and parental survivorship, especially for mothers (Penn and Smith, 2007). On the contrary, since the investment of males in reproduction is usually lower, evolution motivates them toward mating. That could explain why females, compared to males, generally tend to be more sensitive toward babies. Moreover, in an increasingly multicultural society, the investigation of psychological factors that may affect the way people perceive familiar vs unfamiliar individuals (including infants) become pivotal. Both infant and adult faces of in-group and out-group ethnicity are thus stimuli that are presumed to have salient emotional valence, which are probably different in men and women. This study had four hypotheses. First, we expected females to exhibit the Baby Schema effect to both in-group and out-group infant faces. This result would have Baby Schema prevail over the Mere Exposure Effect. Second, taking into account that men show a greater MRE compared to women, we expected an interaction between BS and MRE to be evident in males. Third, considering that individual differences were found in previous infant and adult face processing studies (e.g., Bornstein and Tamis-LeMonda, 1989; Lehmann et al., 2013), we hypothesized that one's neural activation pattern to in-group baby faces and out-group adult faces could be correlated. Past studies have shown that this correlation is gender-dependent. Generally, women, who are known to have higher levels of Baby Schema Effect (e.g., Hess and Polt, 1960; Glocker et al., 2009a), also display higher levels of activation toward out-group faces compared to in-group faces (Richeson and Trawalter, 2008; Trawalter et al., 2008). Fourth, we expected females, but not males, to report no difference in preference between in-group and out-group infant faces, given that females take a greater interest in infants in general and are more inclined in caregiving activities (Maestripieri and Pelka, 2002).

## 2. Study 1

### 2.1. Materials and Methods

#### 2.1.1. Participants

A total of 62 Caucasian Italian adults (31 males, 31 females, Mean Age = 22.55 years, SD = 2.57) were recruited through a database of volunteers available through the University of Trento web site. Informed consent was obtained from all participants, and no incentives were provided. Exclusion criteria were parenthood, pregnancy, and non-Caucasian ethnicity. The study was conducted in accordance with the ethical principles stated in the Helsinki declaration and it was approved by the IRB of the Nanyang Technological University.

#### 2.1.2. Stimuli

Used stimuli were drawn from two different available datasets: asian faces have been selected from Yap et al. (2016), while caucasian faces used in this work have been provided by the Computer Vision Laboratory, University of Ljubljana, Slovenia (Solina et al., 2003; Peer, 2005). Permission has been obtained for the usage of the images. Forty pictures of faces were shown to participants. The faces presented belonged to the following categories that allowed for the manipulation of face age and ethnic group: (i) Baby Caucasian, (ii) Adult Caucasian, (iii) Baby Chinese, (iv) Adult Chinese. Pictures represented neutrally expressive infant and adult female faces (13 × 17 cm) and were obtained from public domain databases. Stimuli were presented in black and white and matched for contrast and brightness using iOS Preview's Tools. Each face was circled within a gray frame to exclude possible distracting information such as hair or background (circle: *d* = 22 cm, area = 380 cm^2^ and circumference = 69 cm). Thus, the stimuli focused on facial feature information.

#### 2.1.3. Experimental Procedure

**Figure 1:**
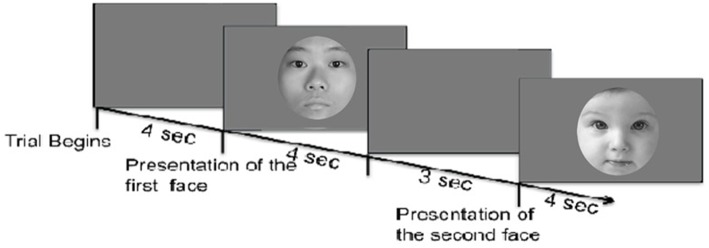
The sketch above illustrates the model representation of the stimuli. Faces were presented in a randomized order across participants. Each face was presented for 4 s followed by an inter-stimulus interval during which a gray screen was shown for 3 s. Face stimuli were selected from Peer (2005) and Yap et al. (2016).

Participants arrived in the laboratory for the experimental session and completed informed consent before starting the experiment. The experiment took place in a quiet darkened environment. We recorded pupil change using an eye-tracking device (Tobii T120, screen: 34 × 27 cm) created by Tobii^1^. After eye tracking calibration, the session started. A within-subjects design was used: each participant saw all pictures in random order. In accordance with previous studies (Bernard et al., 2015; Endendijk et al., 2018), each face was presented for 4 sec followed by a recovery period during which a gray screen, with a central cross served as a fixation point, was displayed for 3 s (Figure 1).

At the conclusion of the procedure, participants completed a questionnaire about their attitude toward each face. All faces were presented once again with three questions aimed to assess participants attitudes: (i) How positive is your attitude toward the face? (ii) How close do you feel to this individual? and (iii) How much do you like this individual? Participants answered each question by moving a sliding cursor on a 0–100 scale, where 0 represented the most negative emotional valence, and 100 represented the most positive emotional valence.

### 2.2. Analysis

#### 2.2.1. Preliminary Analysis

Prior to data analysis, pupil width values were examined for normality, homogeneity of variance, presence of outliers, and influential cases. Outliers, defined as values 2 standard deviations above/below the mean, were log-transformed to make the data conform to normality (Keene, 1995). Average pupil width during the fixation screen preceding each face was considered as a baseline. This baseline measure was subtracted from the average pupil width during the presentation of each face to compute the change in pupil width specific to each face. Change in pupil width for each face was then averaged across the categories of infant and adult in-group and out-group faces (Mathôt, 2018).

Concerning attitudinal responses, participants answers to the three questions for each face category were highly correlated in both genders (females' Attitude and Closeness: Pearson's *r* = 0.852, *p* <2.2e-16; Attitude and Pleasantness: Pearson's *r* = 0.871, *p* < 2.2e-16; Closeness and Pleasantness: Pearson's *r* = 0.947, *p* < 2.2e-16, and males' Attitude and Closeness: Pearson's *r* = 0.931, *p* < 2.2e-16; Attitude and Pleasantness: Pearson's *r* = 0.946, *p* < 2.2e-16; Closeness and Pleasantness: Pearson's *r* = 0.960, *p* < 2.2e-16). Therefore, to take the effect of inter-individual differences on pupil width scores into account, a linear model between pupil width and attitude scores was run. The residuals of the model, representing pupil width changes unexplained by participants' attitude, were used in further analysis.

##### 2.2.1.1. Inferential analysis

Two three-way ANOVA models (one for the left pupil, one for the right pupil) were performed with pupil width residuals as the dependent variable, the two within-subjects factors (face age: baby/adult; ethnic group: Caucasian/Chinese) and one between-subject factor (Sex: Male/Female) as the independent variables. Whenever significant effects emerged, *post-hoc* analyses were carried out by performing Student's *t*-tests. Effect sizes were evaluated using Cohen's d.

### 2.3. Results

#### 2.3.1. Physiological Results

**Figure 2:**
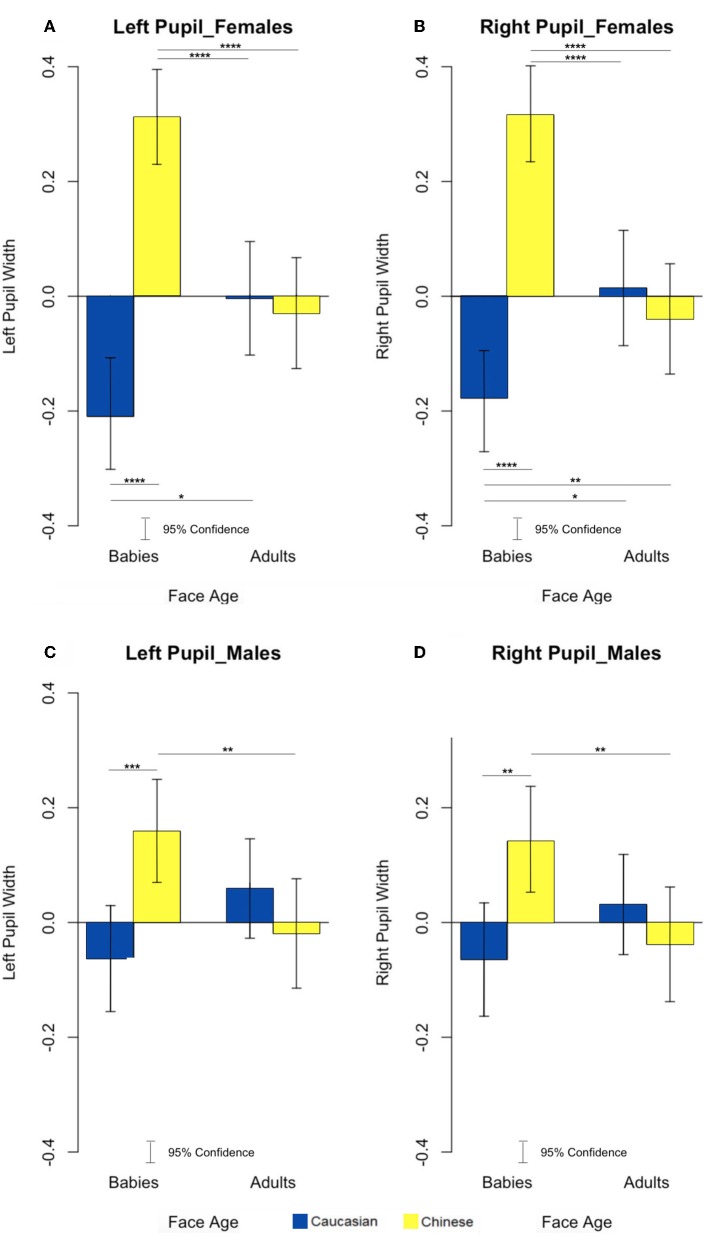
Effects of the interaction between faces age and ethnic group on left and right pupil width in females **(A,B)** and females **(C,D)**. *p < 0.05, **p < 0.01, ***p < 0.001, ****P < 0.0001.

Since it is well-documented that the right and left hemispheres process visual stimuli differently and that this lateralization is also reflected in the pupil variation of the two eyes (Andrew et al., 1982; Hamilton and Vermeire, 1988; Prior et al., 2002; Regolin et al., 2004), we decided to analyze the data separately to verify the presence of any differences in our work. To test hypothesis 1, that females exhibit the Baby Schema effect to both in-group and out-group infant faces, we undertook a 2 × 2 × 2 Analysis of Variance (ANOVA) with age and ethnicity of face as within-subjects factors and gender a between-subject factor; this analysis supported hypothesis 1. After application of a Bonferroni correction to take into account multiple tests, a main effect of ethnic group was found on pupil width of both pupils [left pupils: *F*_(1, 60)_ = 27.49, *p* = 2.157496e-06, η^2^ = 0.0244; right pupils: *F*_(1, 60)_ = 35.53, *p* = 1.430421e-07, η^2^ = 0.0189]. *Post-hoc* analysis revealed that only women increased pupil diameter from baseline toward out-group Chinese faces compared to in-group Caucasian faces [left pupils: *t*_(61)_ = −3.974, *p* = 0.0002, η^2^ = −0.5047; right pupils: *t*_(61)_ = −3.6825, *p* = 0.0005, η^2^ = −0.4677]. To test hypothesis 2, that an association between BS and Mere Exposure Effect (MRE) is evident in males, we observed for interaction effects from the ANOVA models; this analysis partially supports our hypothesis. A significant interaction effect between sex (Males vs. Females) and ethnic group (Caucasian vs. Chinese) of the face was found [left pupils: *F*_(1, 60)_ = 8.33, *p* = 5.400192e-03, η^2^ = 0.0075; right pupils : *F*_(1, 60)_ = 9.82, *p* = 2.673374e-03, η^2^ = 0.0053] and in addition a significant interaction effect between age (Baby vs. Adult) and ethnic group (Caucasian vs. Chinese) of the face was found [left pupils: *F*_(1, 60)_ = 35.62, *p* = 1.385729e-07, η^2^ = 0.0421; right pupils : *F*_(1, 60)_ = 32.53, *p* = 3.815601e-07, η^2^ = 0.0378] (Figure 2).

*Post-hoc* analysis revealed that for both pupils of women and men, there was a significant increase in pupil width in response to Chinese babies faces compared to Caucasian babies faces [left pupils (females): *t*_(30)_ = −6.7712, *p* = 1.652e-07, Cohen's d = −1.0629; right pupils (females): *t*_(30)_ = −6.4622, *p* = 3.861e-07, Cohen's d = −0.9946; left pupils (males): *t*_(30)_ = −4.0984, *p* = 0.0003; Cohen's d = −0.4358; right pupils (males): *t*_(30)_ = −3.4548, *p* = 0.001665, Cohen's d = −0.3799]. In females and males, both pupil widths were significantly wider in response to Chinese baby faces compared to Chinese Adult faces [left pupils (females): *t*_(30)_ = 4.5692, *p* = 7.839e-05, Cohen's d = 0.6774; right pupils (females): *t*_(30)_ = 4.9375, *p* = 2.779e-05, Cohen's d = 0.7047; left pupils (males): *t*_(30)_ = 3.3656, *p* = 0.0021, Cohen's d = 0.3439; right pupils (males): *t*_(30)_ = 3.2478, *p* = 0.0029, Cohen's d = 0.3290]. Only the pupil widths of female participants were significantly reduced in response to Caucasian baby faces compared to Caucasian adult faces [left pupils (females): *t*_(30)_ = −2.5445, *p* = 0.016, Cohen's d = −0.3868; right pupils (females): *t*_(30)_ = −2.581, *p* = 0.0149, Cohen's d = −0.3555]. Pupil widths of female participants significantly increased in response to Chinese baby faces compared to Caucasian Adult faces [left pupils (females): *t*_(30)_ = 4.7998, *p* = 4.098e-05, Cohen's d = 0.6177; right pupils (females): *t*_(30)_ = 4.6619, *p* = 6.042e-05, Cohen's d = 0.5829]. However, only females right pupil significantly decreased in response to Caucasian baby faces compared to Chinese Adult faces [right pupils (females): *t*_(30)_ = −2.8755, *p* = 0.00735, Cohen's d = −0.2618].

#### 2.3.2. Attitudinal Results

**Figure 3:**
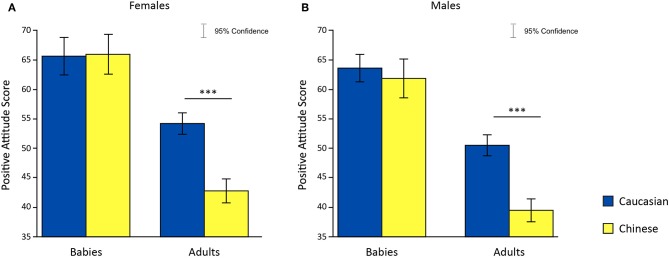
Effects of faces' age and ethnic group on attitude scores in females **(A)** and males **(B)**. *p < 0.05, **p < 0.01, ***p < 0.001.

To test hypothesis 4, that females showed no difference in preference for in-group and out-group infant faces, we undertook a 2 × 2 × 2 Analysis of Variance (ANOVA) with age and ethnicity of face as within-subjects factors, sex of participants as a between-subjects factor and attitude score as the dependent variable; this analysis partially supported our hypothesis. A significant main effect was found for sex [*F*_(1, 60)_ = 8.29, *p* = 5.505286e-03, η^2^ = 4.609139e-02], for ethnic group [*F*_(1, 60)_ = 126.93, *p* = 1.929138e-16, η^2^ = 2.883455e-01] and for age [*F*_(1, 60)_ = 7.84, *p* = 6.855548e-03, η^2^ = 3.850743e-02]. Moreover, a significant interaction between sex of participants and ethnic group was found on attitude scores [*F*_(1, 60)_ = 11.06, *p* = 1.509542e-03, η^2^ = 3.409557e-02] (Figures 3A,B). *Post-hoc* analysis revealed a significant difference in attitude scores in response to adult faces only [females: *t*_(30)_ = 6.06, *p* = 1.1537e-06.001, Cohen's d = 1.1277; males: *t*_(30)_ = 6.29, *p* = 6.1750e-07, Cohen's d = 1.5075], where adult Caucasian faces were rated more positively compared to adult Chinese faces. No significant difference was found between the attitude scores given to Caucasian and Chinese babies faces.

## 3. Study 2

### 3.1. Materials and Methods

#### 3.1.1. Participants

A total of 40 Caucasian Italian adults (20 males, 20 females, Mean Age = 23.175, SD = 2.84) participated. Exclusion criteria were parenthood, pregnancy, and non-Caucasian ethnicity. Informed consent was obtained from all participants prior to the study, and no incentives were given to the participants. This study was conducted in accordance with the ethical principles stated in the Helsinki declaration. Two participants (1 male and 1 female) were omitted from the final analysis because the collected data was corrupted due to technical malfunctions in EEG recordings.

#### 3.1.2. Stimuli

The same set of faces used in Study 1 was shown to participants (refer to section 2.1.2). Similarly, faces were presented for 4 s each in a random order, with a 3-seconds inter-stimulus interval (ISI) between any two faces, as shown in Figure 1. The stimuli were presented using TkInter^2^ on a 29 monitor (DELL 29 Ultrasharp U2719 WM Monitor. Resolution was set to 1,920 × 1,080, refresh rate: 60.00 Hz). Participants were asked to sit approximately 50–70 cm away from the screen. At the end of the presentation, participants completed the same questionnaire employed in Study 1 regarding their disposition toward each face.

#### 3.1.3. Data Acquisition

EEG data were collected using a 14 channel setup (Emotiv EPOC) and digitized at 128 Hz. Previous research has demonstrated the reliability of this device for the recording of EEG signals (Debener et al., 2012; Duvinage et al., 2012; Badcock et al., 2013; De Vos et al., 2014; Ries et al., 2014). E8 of the 14 channels were employed in Study 2: four channels for posterior (P7, P8, 01, 02) and four for anterior areas (F3, F4, F7, F8). Data was recorded on an external device (Lenovo ThinkPad Intel Core i5-4210U).

#### 3.1.4. Preprocessing

Preprocessing of EEG data was implemented using the MNE python package (Gramfort et al., 2013). To reduce the impact of external sources of noise and artifacts, a band-pass filter between 0.51 and 45 Hz was applied to the EEG data. Filtered signals were decomposed into 5 frequency bands—theta (4, 8 Hz), alpha (8, 12 Hz), beta (13, 26 Hz), gamma (26, 45 Hz), and delta (0.51, 4 Hz)—by means of Fast Fourier Transformation. Signals were then normalized, using standard scores, to take into account between- and within-subject differences. Finally, for each stimulus presentation (4 s long), the mean activity and its standard deviation was computed and stored for further analysis. Similar to Study 1, participants answers to the three questionnaires were highly correlated (females' Attitude and Closeness: Pearson's *r* = 0.852, *p* <2.2e-16; Attitude and Pleasantness: Pearson's *r* = 0.870, *p* < 2.2e-16; Closeness and Pleasantness:Pearson's *r* = 0.947, *p* < 2.2e-16 and males' Attitude and Closeness: Pearson's *r* = 0.931, *p* < 2.2e-16; Attitude and Pleasantness: Pearson's *r* = 0.946, *p* < 2.2e-16; Closeness and Pleasantness: Pearson's *r* = 0.960, *p* < 2.2e-16). Therefore, to take into account inter-individual differences, participants attitude toward faces on frequency bands amplitude variations was taken out of the linear model between frequencies amplitudes and attitude scores.

#### 3.1.5. Analytical Plan

##### 3.1.5.1. Preliminary analyses

A repeated-measure mixed design Analysis of Variance (ANOVA) was computed on the 5 frequency bands of selected channels. Amplitude residuals were used as dependent variables. Residual scores controlled for homogeneity (Levene's Test of Equality of Error Variances: Residuals Baby Caucasian *p* = 0.096693, Residuals Baby Chinese *p* = 0.521395, Residuals Adult Caucasian *p* = 0.904724, Residuals Adult Chinese *p* = 0.128961) before residuals were used as the dependent variable in the regression model.

##### 3.1.5.2. Inferential analyses

The first analysis of variance (ANOVA) model tested the effects of gender in moderating BS (hypothesis 1) and MRE (hypothesis 2). Two within-subjects factors (face age: baby/adults; ethnic group: Caucasian/ Chinese) and one between-subject factor (participant gender) were used as independent variables. *Post-hoc* analyses were performed using Student's t-tests. Level of significance was corrected using false discovery rate correction. The second ANOVA model was employed to test whether neural responses to in-group baby faces were related to out-group adult faces (hypothesis 3). We created a new variable to represent the in-group Baby Schema Effect (INBSE) by subtracting the corrected mean amplitudes for the stimuli “adult Caucasian” from the corrected mean amplitudes for the stimuli “baby Caucasian.” A repeated-measures Analysis of Covariance (ANCOVA) was conducted in which this new variable was fitted as a covariate. Face age and ethnic group were used as within-subject factors, and participant gender was used as between-subjects factor. Finally, a repeated-measure mixed design ANOVA was conducted to investigate the impact of face age, ethnic group, and gender on participants attitudes (hypothesis 4). Attitude scores were dependent variables, while the independent variables were kept as the first ANOVA model.

### 3.2. Results

#### 3.2.1. Physiological Results

**Figure 4:**
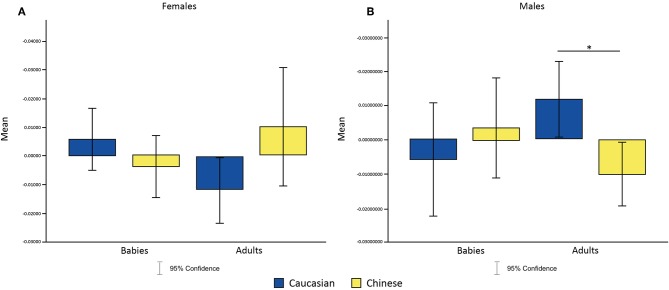
Effects of the interaction between faces' age and ethnic group on P7 alpha amplitude in females **(A)** and males **(B)**. *p < 0.05, **p < 0.01, ***p < 0.001.

To test our first hypothesis, that females exhibit the Baby Schema (BS) effect to both in-group and out-group infant faces, we employed a 2 × 2 × 2 ANOVA where age (Baby vs. Adult) and ethnic group (Caucasian vs. Chinese) of the faces presented were within-subject factors and participant's gender was a between-subject factor; this analysis did not support the hypothesis. An interaction effect between age (Baby vs. Adults), ethnic group (Caucasian vs. Chinese) and participant's gender was found only in P7 channel for alpha wave [*F*_(1, 36)_ = 8.66, *p* = 0.0451040 after Multiple Comparison Correction, η^2^ = 7.280281e-02]. Moreover, *post-hoc* analysis revealed that males showed a significant increase of P7 alpha's amplitude for Caucasian adult faces and a significant decrease in amplitude for Chinese adult faces (*p* = 0.004886, η^2^ = 0.7357) (Figures 4A,B).

**Figure 5:**
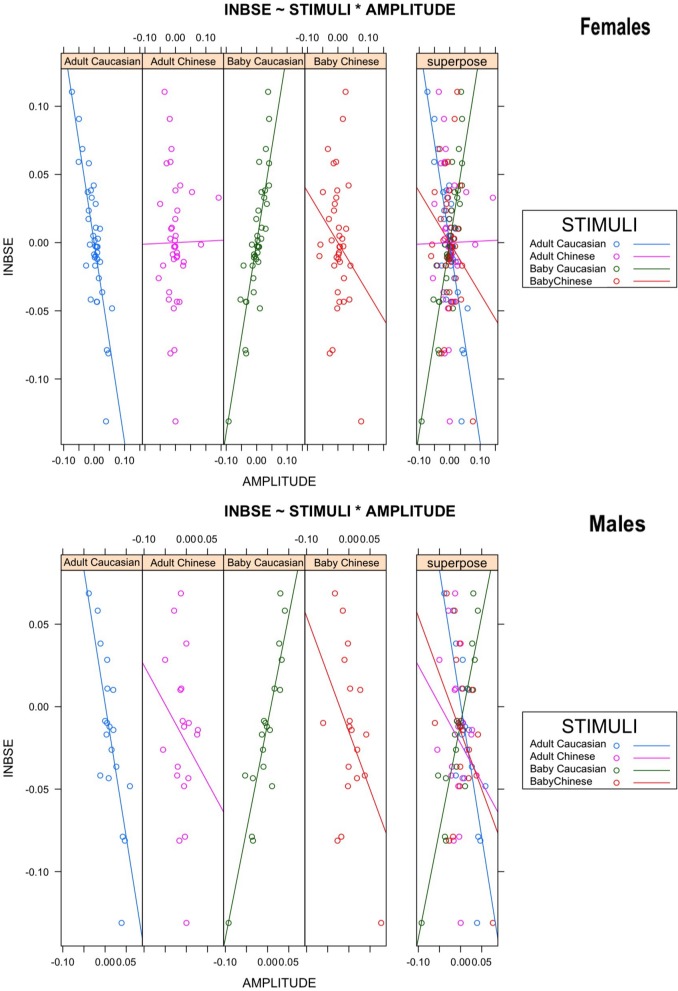
Scatter plots representing the relation between in-group Baby Schema Effect (INBSE) and amplitude of response toward face stimuli in males and females.

To test our third hypothesis, that in-group Baby Schema Effect (INBSE) was related to different activation patterns for out-group stimuli, we undertook a repeated-measures ANCOVA analysis, with INBSE as a covariate, while the age and ethnicity of the face presented were within-subject actors; this analysis supported our hypothesis. A significant three-way interaction between the age of face, the ethnic group of face, and INBSE (*F*_(1, 36)_ = 31,49, corrected *p* = 0.000003, η^2^ = 0.3050] emerged. We also found a significant two-way interaction effect between age of face and INBSE [*F*_(1, 36)_ = 28.88, *p* = 0.000005, after Bonferroni Correction]. *Post-hoc* analysis revealed that participants with higher INBSE showed greater activation toward out-group Adults compared to in-group Adults and that participants with lower INBSE showed greater activation toward in-group Adults compared to out-group Adults (*p* = 0.00015, η^2^ = −0.3062; *p* = 0.000427, η^2^ = 0.2377]. Finally, we found a significant effect of ethnic group of baby faces for lower INBSE (*p* = 0.00015, η^2^ = −0.4292). No main effects were found (Figure 5).

#### 3.2.2. Attitudinal Results

**Figure 6:**
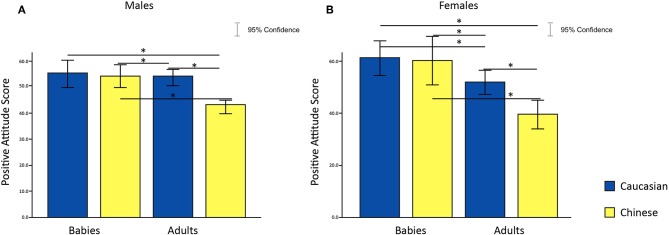
Effects of faces' age and ethnic group on attitude scores in females **(A)** and males **(B)**. *p < 0.05, **p < 0.01, ***p < 0.001.

To test our fourth hypothesis, that preference for in-group and out-group infant faces do not differ for females, we conducted a 2 × 2 × 2 ANOVA with age and ethnicity of face as within-subjects factors, sex of participants as a between-subjects factor and attitude scores as the dependent variable; this analysis partially supported our hypothesis. From the analysis, we found main effects of age and ethnic group of a face [age: *F*_(1, 36)_ = 43.32, *p* = 1.1683E-7, η^2^ = 0.1804; ethnic Group: *F*_(1, 36)_ = 26.20, *p* = 1.044176e-05,η^2^ = 0.0705]. We also found a significant interaction between age and ethnic group of the face on attitude scores for both males and females [*F*_(1, 36)_ = 46.03, *p* = 6.3027E-8, η^2^ = 0.0527]. Moreover, we found a significant age of face and sex of participant interaction, meaning that males and females treated baby and adult faces differently [*F*_(1, 36)_ = 7.81, *p* = 0.008, η^2^ = 0.0382]. *Post-hoc* analysis revealed that Caucasian baby faces evoked greater positive attitudes compared to Chinese adult faces in both genders [females: *t*_(18)_ = 7.112, *p* = 0.000001, Cohen's d = 1.753; males: *t*_(18)_ = 5.386413, *p* = 0.00004, Cohen's d = 1.390]. Only in females was there a greater preference for Caucasian baby faces compared to Caucasian adult faces [females: *t*_(18)_ = 3.604883, *p* = 0.002, Cohen's d = 0.8181]. Both genders showed more positive attitudes toward Caucasian adult faces compared to Chinese adult faces [females: *t*_(18)_ = 6.809751, *p* = 0.000002, Cohen's d = 1.109; males: *t*_(18)_ = 7.523347, *p* = 5.812E-7, Cohen's d = 1.733], and Chinese baby faces compared to Chinese adult faces [Females: *t*_(18)_ = 6.610146, *p* = 0.000003, Cohen's d = 1.323; Males: *t*_(18)_ = 4.834543, *p* = 0.00013, Cohen's d = 1.109]. No significant difference in attitude scores was found between baby faces stimuli (Figures 6A,B).

## 4. Discussion

This study investigates the Baby Schema (BS) and Mere Exposure (MRE) effects on adults implicit and explicit responses to infant and adult in-group and out-group faces. Baby Schema is a collection of infant facial features which has been selected by evolution to inspire caregiving behaviors in adults. MRE dictates that familiarity leads to preference, which begets the question of whether the protective effect of BS applies to both in-group (familiar) and out-group (unfamiliar) infants. We investigated Italian adults implicit physiological responses using pupillometry and electroencephalography while they were presented with in-group (Caucasian) and out-group (Chinese) infant and adult faces. Questionnaire data were also collected to obtain measures of participants explicit self-reported preferences of faces.

Results from Study 1 revealed several key findings. First, females exhibited greater pupillary dilation to out-group infants than out-group adults (Chinese baby >Chinese adult) and greater pupillary constriction to in-group infants than in-group adults (Caucasian baby >Caucasian adult) in both pupils. Two cross-ethnic group comparisons (i.e., Chinese baby >Caucasian adult; Caucasian baby >Chinese adult) were observed in the right pupil while only one was observed in the left pupil (i.e., Chinese baby >Caucasian adult). Males showed a BS effect in both pupils specific to out-group Chinese infants (i.e., Chinese baby >Chinese adult), thus partially supporting hypothesis 1.

From Study 2, we found greater left posterior-parietal alpha activation toward familiar in-group (i.e., Caucasian) adult faces compared to unfamiliar out-group (i.e., Chinese) adult faces in males. Since alpha activation is inversely related to neural activity, this shows that males exhibit more activation and thus attention toward out-group faces, which suggests the presence of MRE and supports hypothesis 2. We conducted another analysis by stratifying participants based on their in-group Baby Schema effect (INBSE) score, from which we found that participants lower in INBSE showed significantly different activation patterns between Chinese and Caucasian baby faces, whereas participants higher in INBSE did not. Instead, those with a higher INBSE score exhibited distinct activation between Chinese and Caucasian adult faces, which indicates that those with higher INBSE differentiated between in-group and out-group adults at the early stage of face processing. These findings suggest that level of INBSE moderates MRE for adult and baby faces, which supports hypothesis 3.

Questionnaire responses from participants, which was an explicit measure of self-reported preference of faces, were consistent in both Study 1 and 2. Males and females reported greater preference for in-group adult faces, which suggests an MRE specific to adult faces. Participants in Study 2 showed greater preference for baby over adult faces which reflects an overt BS effect. However, gender was not found to moderate preferences for baby faces as expected in hypothesis 4.

### 4.1. Gender Moderates Baby Schema and Mere Exposure Effects

Overall, we found in females a greater pupillary response toward children than adults. However, looking specifically at the direction of the variations, we observed that this response goes in two different directions: a pupil dilation toward out-group infants and a pupil constriction toward in-group ones. Regarding pupil dilation, past studies (Glocker et al., 2009a,b), where variables such as in-group and out-group were not taken into account and where the physiological measure was for example the neural response, highlighted that women generally showed greater responses toward infants. Therefore, it is possible to assume that the dilation that we observed in our study, which in general is a synonym for more attention given to stimuli (Aktar et al., 2016), means more attention given to infants than to adults. Hess and Polt (1960) similarly observed larger pupil dilation in women looking at pictures of infants than of other people, lending further support to our findings. Instead, the observed pupil constriction for in-group babies is more challenging to explain, considering the non univocal origin of pupil constriction (Loewenfeld, 1966; Hess, 1972; Mathôt et al., 2013; Mathôt and Van der Stigchel, 2015; Winn et al., 2018; Zekveld et al., 2018). However, in line with this result, Landi and Freiwald (2017) found that a greater pupillary constriction occurs in response to familiar faces compared to unfamiliar ones, suggesting that familiar faces request fewer cognitive resources in order to be processed. Therefore, the picture that emerges is that both BS and MRE are active in females while they process faces of different ages and ethnicity. This co-existence leads them to show more attention to infant faces than adult faces but with opposite directions for Caucasian and Chinese faces due to the familiarity of the stimuli.

By comparison, the BS effect elicited in males was specific to out-group faces only, which is in line with our second hypothesis, that an interaction effect between BS and MRE is likely to be observed in males. Pupil dilation indicates greater preferential attention and has been thought to occur during an increase in processing effort (Kahneman, 1973; Beatty, 1982). As compared to familiar in-group faces, unfamiliar out-group faces are more novel and evoke greater attention. The processing of unfamiliar out-group faces might require the recruitment of more cognitive resources (Goldinger et al., 2009; Hu et al., 2014). Therefore, the BS effect which entices adults to attend to infant faces might have been moderated by the MRE effect which induces enhanced attention to out-group faces, leading to significantly greater pupillary dilation in males toward baby than adult faces for out-groups.

While gender difference in regard to the Baby Schema effect is well-established (Hess and Polt, 1960; Glocker et al., 2009a), we found that gender also moderates MRE. Males in Study 2 exhibited amplitude reduction of the alpha band, corresponding to neural activation, in the left posterior-parietal region toward unfamiliar out-group (Chinese) adult faces when contrasted against familiar in-group (Caucasian) adult faces. From an evolutionary point of view, better processing of new and potentially threatening stimuli in the environment (out-group faces) is pivotal and may guarantee the individual's survival. Amplitude decrease of alpha indicates a state of relatively high excitability and engagement of the brain area, while an amplitude increase reflects inhibition (Hart et al., 2000; Ito and Urland, 2005; He et al., 2009). Posterior parietal areas are involved in attentional processing (Buschman and Miller, 2007) and activity in the alpha system may predict attention toward threatening social stimuli (Yamakawa et al., 2009; Giardina et al., 2012; Grimshaw et al., 2014). We might deduce therefore that males attention is more effectively captured by out-group adults, possibly due to evolutionary-enhanced processing which occurs in response to unfamiliar stimuli that may pose a threat. Behavioral reports from Study 1 and 2 showed greater preference toward in-group adult faces, suggesting that MRE is present at an explicit level in both males and females (Allport et al., 1954; LeVine and Campbell, 1972; Brewer, 1979; Esposito et al., 2014).

Attitudes toward infant and adult faces varied across the two studies. Participants in Study 1 showed no difference in attitude toward infant and adult faces, but both male and female participants in Study 2 reported more positive attitudes to infant faces compared to adult faces. These reports show the existence of conscious perception of the BS effect (Glocker et al., 2009a,b; Parsons et al., 2011). However, we did not find any difference in reports of preference for infant compared to adult faces between male and female participants as we initially hypothesized.

### 4.2. Baby Schema Moderates Mere Exposure Effect

Individuals who experience the BS effect have been found to possess higher levels of empathy, interpersonal closeness, and need to belong (Lehmann et al., 2013). These personality traits might also influence implicit perceptions toward in-group and out-group faces. To investigate this relation, we stratified our participants according to their Baby Schema score. We found that participants with lower BS scores differed in their processing of in-group and out-group infant faces which fulfilled our third hypothesis. A possible interpretation is that participants with lower BS scores are less susceptible to the BS effect such that they discriminate between in-group and out-group infants. Participants with higher BS scores are generally more prone to find infant faces appealing and are less likely to process in-group and out-group infant faces differently. Similar neural processing of infant faces of different ethnicity also suggests some protective effect of BS when perceiving unfamiliar out-group infants (Proverbio and De Gabriele, 2017).

Higher BS scores were associated with reduced alpha amplitudes (increase in neural activation) in response to out-group adult than in-group adult faces. This suggests that a strong BS effect, in which no difference is detected in the processing of in-group and out-group infants is potentially indicative of substantial MRE effects among adults (Proverbio et al., 2011; Proverbio and De Gabriele, 2017). It is reasonable to assume that participants who are more interested in caregiving activities are also more sensitive to potentially threatening stimuli to infants, such as unfamiliar out-group adults (Lorenz, 1943; Lorenz and Martin, 1971; Glocker et al., 2009a,b).

### 4.3. Limitations

Here we report some limitations and propose possible future directions of research. First, the main limitation of the present work concerns the absence in the available literature of a unequivocal interpretation of the pupil constriction toward in-group baby faces observed in the pupillometry study. However, this result is in line with (Landi and Freiwald, 2017) who found that familiar faces elicit greater pupillary constrictions compared to unfamiliar ones. The simultaneous presence of MRE and the BS might explain why in females more attention is given to infants than adults but with opposite direction depending on the familiarity (Caucasian vs. Chinese). To confirm this interpretation, however, a future study should try to test the same paradigm in a different population where Chinese faces are more familiar than Caucasian faces. Second, this study utilized neutral faces only, and future studies should assess the effect of emotional valence in facial expressions (i.e., smiling, crying). Third, consistent with other studies (e.g., Esposito et al., 2015), only two ethnic groups were examined. Employing a range of different ethnic groups (e.g., African, Latin Americans, et al.) could better expose relations between social distance from out-groups and face processing. Fourth, only adult female faces were used; it has been well-established that males and females react differently to faces of adult women. This gender bias could be overcome by including adult male faces in the stimuli. Fifth, future studies may combine frequency bands analysis (neural oscillations) of face processing with other existing measures of EEG, like Event Related Potential (ERP). ERP analysis was not conducted in this study as it would have required multiple repetitions of each stimulus that would not have enabled us to be consistent with the pupillometry study. Sixth, despite the fact that we analyzed data collected from a generally individualistic society, it may be also interesting to replicate this study in an more collectivist society to see how culture shapes faces perception. Finally, future investigators should also consider individually held attitudes and experiences with infants as well as out-group members.

## 5. Conclusion

The human face contains tremendous social information and plays a fundamental role in communication with other people. Our study has revealed the rich interplay of Baby Schema (BS) and Mere Exposure (MRE) effects that emerge on merely viewing adult and infant faces. We showed that gender greatly moderates the processing of faces. Given the simultaneous presence of both MRE and BS, females pay more attention to infant than adult faces but the responses are opposite for Caucasian and Chinese faces (constriction vs. dilation) due to the familiarity of the stimuli. By comparison, males attend to out-group infants significantly more than out-group adults, but require more cognitive recruitment to process out-group than in-group adult faces. While gender differences were observed at the implicit processing level, males and females did not differ in their self-reported attitudes, as both reported greater preference for infant over adult faces. Contradictory results that emerge at the implicit (physiological) and explicit (self-report) levels indicate that differential processing of faces varying in age and ethnicity may occur at a non-conscious level. Besides gender, level of Baby Schema effect may reflect empathy and predict differential processing of adult and infant faces belonging to an individual's in-group or out-group. These findings evoke excitement regarding the role of gender and personality in moderating face processing. Ingrained in us is a complex biological attentional mechanism that extracts critical information from the humble face and elicits automatic responses that have been shaped by evolution and society.

## Data Availability Statement

The dataset Esposito et al. (2019) generated for this study can be found in the NTU's Data repository DR-NTU Data at the following address: https://doi.org/10.21979/N9/TGTTTR.

## Ethics Statement

The studies involving human participants were reviewed and approved by the IRB of the Nanyang Technological University. The patients/participants provided their written informed consent to participate in this study.

## Author Contributions

LV, MB, and GE conceptualized the study. LV collected the data. LV, GG, and AT analyzed the data. LV and AA wrote the original draft. All the authors reviewed and edited the submitted version of the article.

### Conflict of Interest

The authors declare that the research was conducted in the absence of any commercial or financial relationships that could be construed as a potential conflict of interest.

## Footnotes

^1^https://www.tobii.com

^2^https://wiki.python.org/moin/TkInter
